# Facial emotion processing hemispheric bias is weakly associated with handedness, autistic traits and biological sex, but not age

**DOI:** 10.1186/s40359-024-02218-2

**Published:** 2025-03-18

**Authors:** B. E. Speranza, M. Do, A. T. Hill, P. H. Donaldson, P. G. Enticott, M. Kirkovski

**Affiliations:** 1 Cognitive Neuroscience Unit, School of Psychology, Deakin University, Burwood, VIC Australia; 2 Institute for Health and Sport, Victoria University, Melbourne, Australia

**Keywords:** Hemispheric specialisation, Hemispheric laterality, Hemispheric asymmetry, Autistic traits, Autism, Right-hemispheric bias, Handedness, Emotion processing, Social cognition

## Abstract

**Background:**

Right-hemisphere brain regions are strongly implicated in facial emotion processing (FEP), a phenomenon termed right-hemispheric bias. Variability in FEP hemispheric bias is thought to underpin differences in facial emotion recognition ability and has been associated with age, handedness, biological sex, and autistic traits. However, findings from research to date investigating factors associated with FEP hemispheric bias have been inconsistent.

**Objective:**

To examine if FEP hemispheric bias can be predicted by individual factors such as age, biological sex, handedness, and autistic traits.

**Methods:**

427 adults recruited from the general population aged 18–67 years completed the Autism-spectrum Quotient. We also assessed covariates previously linked with FEP hemispheric bias including age, handedness, and biological sex. FEP hemispheric bias was indexed using laterality quotients calculated from a Chimeric Faces Task, where participants indicated which of two identical (but mirrored) half-emotional half-neutral (no emotion) chimeric faces were more emotive.

**Results:**

Linear regression models revealed that (1) handedness predicted FEP hemispheric choice bias, (2) the attention switching Autism-spectrum Quotient subscale predicted FEP hemispheric reaction time bias, and (3) the imagination Autism-spectrum Quotient subscale predicted FEP hemispheric reaction time bias for males, but not females.

**Conclusions:**

These findings indicate that the relationship between autistic traits and FEP hemispheric bias is nuanced. Additionally, handedness influences hemispheric bias effects during FEP. Future research should endeavour to investigate if FEP hemispheric bias is dependent on the emotion being observed and consider using more direct measures of hemispheric bias.

**Supplementary Information:**

The online version contains supplementary material available at 10.1186/s40359-024-02218-2.

## Highlights

Right-handed people are more likely to exhibit a right-hemispheric bias during facial emotion processing than left-handed people.

Levels of autistic traits related to attention switching may predict hemispheric bias during facial emotion processing.

Levels of autistic traits related to imagination may predict hemispheric bias during facial emotion processing for males, but not females.

The relationship between relevant demographic and individual characteristics and hemispheric bias during facial emotion processing is nuanced and may be affected by other factors that warrant investigation.

Future research should utilise large, diverse samples, and employ various methodological approaches to ensure comprehensive investigation.

Recognising and understanding facial emotions (referred to as facial emotion processing; FEP) is vital for interpersonal communication and navigating daily social interactions [1, 2]. FEP is strongly lateralised to regions in the right hemisphere of the brain, a phenomenon termed right-hemispheric bias [RHB; 3, 4, 5]. However, the extent to which one exhibits a RHB varies between individuals, with some people having a stronger RHB than others and, although less common, some people being left lateralised [3, 4, 6]. Atypical patterns of hemispheric bias during FEP have previously been observed in individuals diagnosed with conditions characterised by FEP difficulties, namely, autism spectrum disorder [7–9]. Some research suggests that the strength and direction of hemispheric bias during FEP may underpin the common emotion recognition difficulties experienced by autistic people [7–9]. However, findings regarding FEP hemispheric bias in autistic cohorts are mixed. Specifically, some research suggests that autistic individuals exhibit an atypical left hemispheric bias (LHB) for FEP of happy, sad, and angry emotional faces [8]. Other research, however, has demonstrated that autistic people’s hemispheric bias for FEP of angry and happy emotional faces does not differ to that of neurotypical individuals [7]. Similarly, Taylor et al. [9] found that autistic children exhibited a RHB for happy and angry faces, but no hemispheric bias was observed for sad, surprised, disgusted, or fearful faces. Notably, these studies relied on small sample sizes (*n* = 18 to *n* = 32), and Brindley and Schmidt [8] and Taylor et al. [9] investigated this effect in children. The inconsistent findings, and heterogenous methodology of previous literature, highlights the need for further investigation, in a larger sample, exploring how autistic traits are implicated in the processing of emotional faces.

Levels of sub-clinical autistic traits might underly FEP hemispheric bias differences seen in neurotypical individuals [10]. Vladeanu et al. [10] found individuals with higher scores on a measure related to social interest (indicating higher autistic traits) had a stronger RHB than those with lower scores. This was, however, only true for male participants, while the reverse was found for females [higher autistic traits associated with social interest were related to weaker RHB;^10^]. The notion that levels of specific autistic traits may be related to FEP hemispheric bias has not yet been elucidated. Additionally, the difference between male and female participants was only evident for fear, happiness, and surprise [10]. This suggests that there may also be factors such as sex differences, underpinning hemispheric bias during FEP.

As well as biological sex [10, 11] evidence suggests there are numerous other individual factors that may underly FEP hemispheric bias differences, such as age [11, 12] and handedness [13–15]. Specifically, research suggests that older people [12, 16], left-handed people [13, 15], and females [11, 13] tend to be less right lateralised for FEP. There are differences observed in emotion recognition among these groups which may be related to FEP hemispheric bias, but these differences are inconsistent [10, 17–20]. Older people tend to be less accurate when identifying facial emotions [18, 21], while women have been observed to respond quicker and more accurately [20]. These findings call into question the nature of the relationship between emotion recognition and FEP hemispheric bias, as the direction of the effect appears to differ between demographic groups. Conversely, some research reports no age [19], sex [17], nor handedness [10, 17] effects on hemispheric bias for FEP. Importantly, even when no significant effect of sex was reported, a trend aligning with previous research (i.e., females exhibiting reduced RHB for FEP than males) was observed [17]. Further, research reporting no age effect on FEP hemispheric bias only investigated this effect in females [19]. Given that some research suggests females are less right lateralised for FEP than males [11], this could explain the null findings reported. Finally, handedness is known to be related to the hemispheric asymmetry of several cognitive processes, including language, vision, and working memory [22]. A meta-analysis reported that autistic individuals are more likely to be (a) non-right-handed, (b) left-handed, or (c) mixed-handed when compared to typically developing individuals [23]. The importance of handedness in FEP hemispheric bias, and how this may relate to levels of autistic traits is not yet clear. Previous research investigating the relationship between handedness and FEP hemispheric bias has consistently only found a significant relationship when handedness was investigated categorically [13–15], but not when investigated continuously [10, 17]. The pattern of inconsistency in the findings of research investigating the relationship between (1) FEP hemispheric bias and emotion recognition ability, and (2) individual variability and FEP hemispheric bias highlights the need for more thorough investigation. In order to examine these relationships, it is important to first elucidate the underlying factors involved in these processes. Disentangling the factors affecting hemispheric bias during FEP will offer insight into the neural processing of facial emotion stimuli, informing future research seeking to investigate this process in both clinical and non-clinical settings.

Previous research has been characterised by small sample sizes, contributing to findings exhibiting significant heterogeneity. Additionally, research to date has employed varying methodological approaches, which may further confound research findings. Finally, findings from existing research investigating these factors in isolation is inconsistent and has neglected to consider the interplay between these factors in FEP hemispheric bias. This has led to difficulty both interpreting and generalising findings, and elucidating the role various individual factors play in FEP. The present research sought to address this by employing a large and diverse sample from the general population to facilitate a more robust and comprehensive understanding of the role age, biological sex, handedness, and autistic traits play in FEP hemispheric bias. Further, research investigating the role of autistic traits in FEP hemispheric bias is scarce and tends not to investigate the nuanced relationship between autistic traits and FEP, and instead often takes a categorical diagnostic approach. This research will build on the current body of knowledge by investigating how traits associated with autism [via a broad autism phenotype approach; 24] are related to FEP hemispheric bias. This could inform development of future clinical intervention/resources for those seeking support for social and communication symptoms which is a core feature of autism.

The present research will aid in understanding the predictive ability of individual factors such as age, biological sex, handedness, and autistic traits for hemispheric bias during FEP. How these individual factors combine to explain FEP hemispheric bias has not been examined thus a thorough investigation of the factors together is necessary. This knowledge will inform future research seeking to investigate FEP, and the relationship between FEP hemispheric bias and FEP ability. Elucidating the role of these factors will aid in identification of those who may be at an elevated risk of experiencing FEP difficulties allowing support via early intervention. This is particularly relevant for individuals diagnosed with autism, who may see their diagnosis negatively impacting their interpersonal relationships due to the effect of their diagnosis on social cognition/emotion processing. To this end the current study used a behavioural approach to investigate the relationship between FEP hemispheric bias and age, biological sex, handedness, and autistic traits in a large sample from the general population. Specifically, this research sought to examine the unique relationship between individual factors including age, biological sex, handedness, and autistic traits, and hemispheric bias during FEP. With previous research in mind, it was hypothesised that older people, left-handed people, and females would exhibit a reduced RHB for FEP than younger people, right-handed people, and males, respectively. It was also hypothesized that individuals demonstrating higher levels of social and communication related autistic traits, as measured by the Autism-spectrum Quotient (AQ), would exhibit reduced RHB for FEP.

## Methods

### Participants

This study was approved by the human research ethics committee of Deakin University (HEAG-H 187_2021), and in accordance with the Declaration of Helsinki. Informed consent was obtained from all participants.

517 adult participants from the general population were recruited via the online crowd sourcing platform Prolific [25]. Of these, 427 participants (214 female, 210 male, and 3 did not disclose their biological sex) were included for data analysis (see “Data Cleaning” for more information). Demographics for included participants are presented in Table 1. Participants were reimbursed approximately GBP£3.78 upon study completion.

**Table 1:** Participant characteristics

Demographics				
Continuous variables	M	SD	Min	Max
Age (years)	27.23	8.43	18	67
EHI score	77.83	55.17	-100	100
AQ score	114.4	14.72	70	159
Categorical variables	N	%		
Biological sex				
Female	214	50		
Male	210	49		
Not reported	3	< 1		
Self-reported handedness				
Right	378	89		
Left	39	9		
Ambidextrous	10	2		
Education level (highest obtained)				
Primary Education	4	< 1		
Secondary Education	69	16		
Certificate Level	47	11		
Graduate Diploma or Graduate Certificate Level	58	14		
Advanced Diploma or Diploma Level	30	7		
Bachelor Degree Level	184	43		
Postgraduate Degree Level	32	7		
Other Education	3	< 1		
Continent of Residence				
Africa	90	21		
Asia	9	2		
Europe	111	26		
North America	164	38		
Oceania	15	4		
South America	13	3		
Prefer Not to Say	25	6		

Note.*N* = 427. AQ = Autism-spectrum Quotient; EHI = Edinburgh Handedness Inventory

### Materials and procedures

#### Demographic measures

Participants were first asked a series of questions regarding their basic demographics (including age, handedness, and biological sex) in the online survey distribution tool Qualtrics [26 Provo, UT]. In addition to self-reported handedness, participants completed the Edinburgh Handedness Inventory [EHI; 27].The EHI is a 10-item questionnaire, measured on a 5-point scale, designed to determine hand preference. Scores range from − 100 to 100 with negative scores indicating left-hand preference, and positive scores indicating right-hand preference. The absolute value represents preference strength, with scores near 0 reflecting no preference.

#### Autistic traits

Following the demographics questionnaire and EHI, participants completed the Autism-spectrum Quotient [AQ; 28], also in Qualtrics (Qualtrics, Provo, UT). The AQ is a 50-item self-report questionnaire, measured on a 4-point scale, designed to assess traits and characteristics associated with autism in the typically developing population. The AQ is comprised of five subscales reflecting symptoms frequently associated with autism: social skills, attention switching, attention to detail, communication, and imagination. While originally scored on a 2-point scale, here AQ scores were calculated using the alternative method, employing a 4-point scale (Lundqvist & Lindner, 2017). Scores range from 50 to 200, where higher scores reflect higher autistic traits. Within the present sample, moderate to high internal consistency was noted across each of the five subscales of the AQ (communication; α = 0.72, social skills; α = 0.77, imagination; α = 0.50, attention to detail; α = 0.70, and attention switching; α = 0.65).

#### Facial emotion processing hemispheric bias

**Fig. 1:**
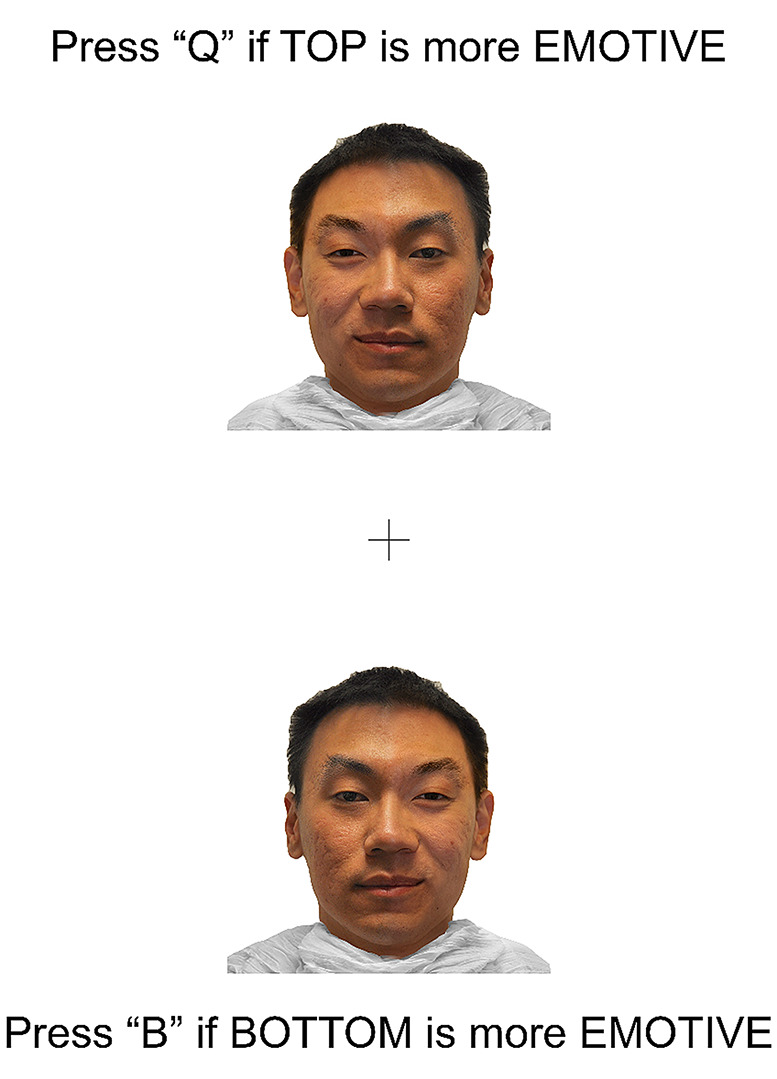
*Example trial image. Note.* Example of a given trial image presented to participants. In this example, if participants chose the top image (which contains the emotion in the left visual field) this would demonstrate a right hemispheric bias for facial emotion processing. Conversely, choosing the bottom face (with the emotion in the right visual field) would indicate a left hemispheric bias for facial emotion processing

Lastly, participants completed a Chimeric Faces Task in the psychological testing software Inquisit [29] designed to assess hemispheric bias of FEP. Stimuli from this task were modified from the racially diverse affective expression (RADIATE) face stimulus set [30, 31]. During this task, participants were presented with images of chimeric faces (i.e., photographs of faces which have been split vertically down the midline, to present an emotion [happy, angry, or fearful] on one side of the photographed face, and a neutral expression [no emotion] on the other). Each chimeric face is mirrored horizontally, to create a new chimeric face that is identical to the first, but with the emotional and neutral hemifaces reversed. These images were then presented to the participant simultaneously, one above the other. This display is the trial image. Two versions of each trial image were generated and presented, one with the face depicting the emotion in the left visual field at the top, and one with this image at the bottom, to control for confounds associated with vertical image placement on the screen. Figure 1 shows an example of a single trial image.

**Fig. 2:**
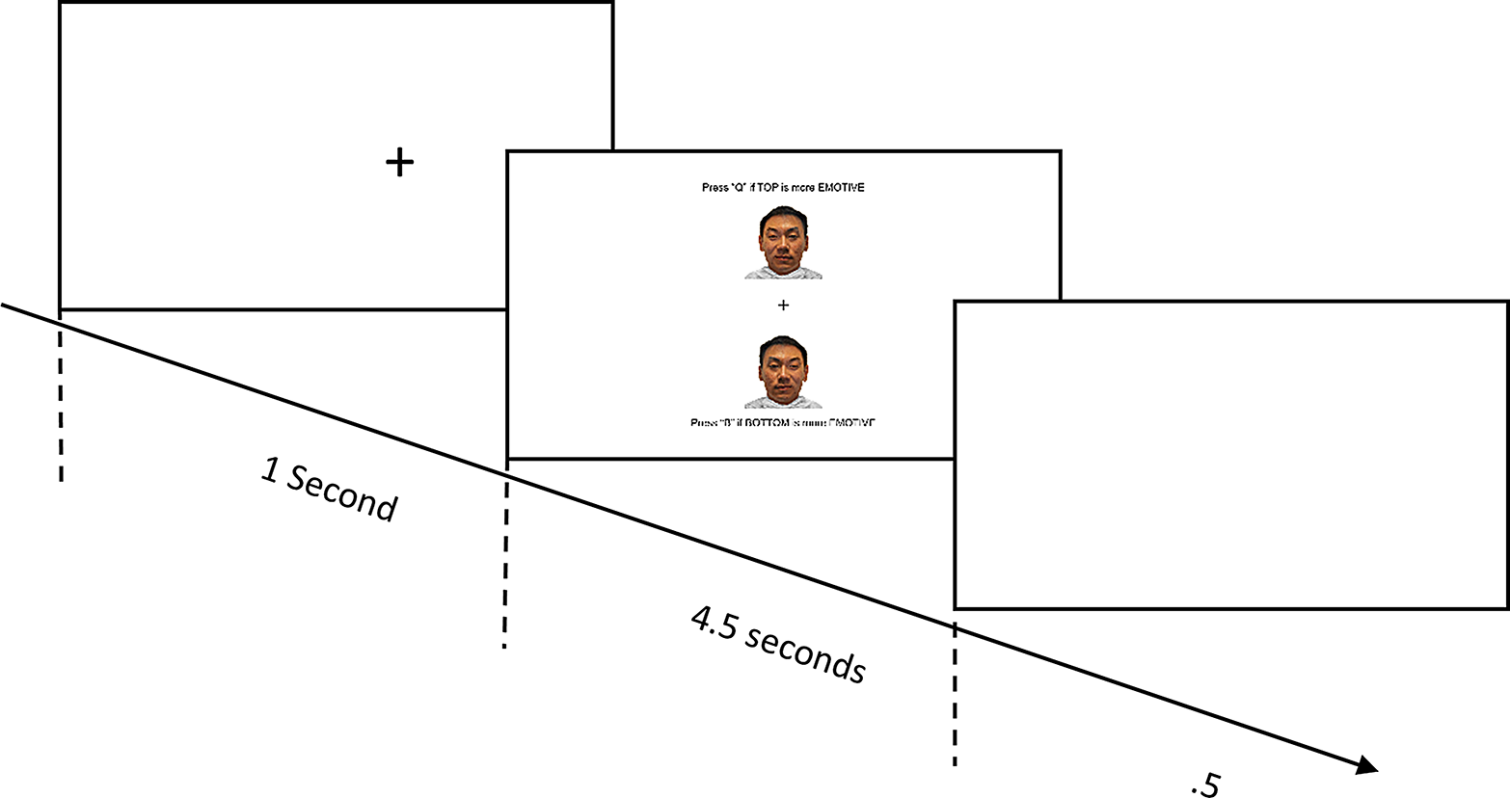
Chimeric Faces Task Paradigm. Note.Each individual trial consisted of a fixation cross presented for 1 s, followed by the trial image presented until either (**a**) the participant made a response, or (**b**) 4.5 s had passed. This was followed by a 0.5 s inter-trial interval. Following this the next trial began

Each individual trial consisted of a fixation cross presented for 1 s, followed by the trial image presented until either (a) the participant made a response, or (b) 4.5 s had passed. This was followed by a 0.5 s inter-trial interval. The task consisted of 192 trials (excluding practice and attention check trials; emotions [happy, angry, fearful] equally distributed across the trials). Participants were required to indicate via keyboard button press (“Q” for the top image, “B” for the bottom image), as quickly as possible, which of the two images they perceived to be more emotionally intense. Choosing the image with the emotion in the left visual field demonstrates a RHB for FEP, while choosing the image with the emotion in the right visual field demonstrates a LHB for FEP. Following this, participants were presented with a new fixation cross, followed by the next trial. The order of the trial images was randomised for each participant. Figure 2 shows the sequence of trial stimuli.

The Chimeric Faces Task has been previously validated as a reliable tool for measuring hemispheric bias during FEP [32]. Additionally, use of the Chimeric Faces Tasks in assessing hemispheric bias during FEP has been validated in studies investigating its use among both adults [33] and children [34] with unilateral left and right hemispheric lesions. Specifically, those with damage to the right hemisphere exhibited reduced RHB compared to controls and those with left hemisphere damage [33, 34].

Two hemispheric bias scores (laterality quotients; LQ) were calculated. The first, LQ1, was based on the number of trials in which the participant chose the face that had the emotion presented in the left visual field, vs. the right visual field, using the formula:

$$\:\frac{\left({N}_{L}-{N}_{R}\right)}{{N}_{Total}}$$

Where N_L_ is the number of trials in which the face with the emotion in the left visual field was chosen, N_R_ is the number of trials in which the face with the emotion in the right visual field was chosen, and N_total_ is the total number of trials. The index score ranges from − 1 to + 1, where positive scores indicate a left visual field/right hemisphere advantage, and negative scores indicate a right visual field/left hemisphere advantage. This method of generating an LQ has also been used in previous research [7, 17] and has been validated, as discussed earlier [32–34].

The second, LQ2, was based on reaction time when the participant chose the face with the emotion in the left visual field, vs. the right visual field, using the following formula:

$$\:\frac{\left(R{T}_{R}-R{T}_{L}\right)}{R{T}_{Total}}$$

Where RT_R_ is the average reaction time for trials in which the face with the emotion in the right visual field was chosen, RT_L_ is the average reaction time for trials in which the face with the emotion in the left visual field was chosen, and RT_Total_ is the average reaction time across all valid trials. Similar to LQ1, the index score ranges from − 1 to + 1, where positive scores indicate a left visual field/right hemisphere advantage, and negative scores indicate a right visual field/left hemisphere advantage. Research suggests the inclusion of reaction time based LQs may offer more information regarding hemispheric bias and has been strongly correlated with the traditional LQ index [i.e., LQ1; 17]. In previous research raw differences in reaction time were taken to indicate hemispheric bias [17]. Here, we extended this method to account for individual participant’s total reaction time.

### Data cleaning

Data cleaning consisted of removal of participants who (a) did not complete the questions/tasks in full, (b) did not pass a set number of attention checks, (c) did not pass a free text entry bot check, or (d) exhibited an unusual pattern of responses. Additionally, due to the low number of participants who did not specify their sex these participants were excluded from data analyses that included sex as a factor. Methods for data cleaning are outlined below.

#### Attention checks

2–4 attention checks occurred randomly throughout the Chimeric Faces Task. In these attention check items participants were presented with two faces in the same format as a standard trial, however, instead of chimeric faces, one face was neutral (no emotion), and the other presented an emotion. These trials asked participants to select a specific keyboard entry. As an example, the text for one these trials read “This is an attention check. Please Press ‘Q’”. In addition, to assess participant attention to instructions, at the conclusion of the task participants were asked (via a multiple-choice response) what the task required them to do. An additional attention check was presented during the AQ. This attention check item read “This question just checks your attention. Please select the option “slightly disagree” for this question (ignore all other options).“. In total participants completed at least 4 attention checks (some pilot participants completed a total of 6 attention checks; other than some additional attention checks, there were no differences between pilot and non-pilot methodology). Participants who failed 2 or more attention checks (or 3 or more for pilot participants who received more attention checks) were excluded from data analyses.

#### Bot check

A bot check was presented at the end of the Chimeric Faces Task. Participants were required to describe what they saw in an image, using free-text entry. Participants whose responses did not align with the image were excluded from data analysis.

#### Response patterns

Participant responses were manually reviewed for unusual response patterns (e.g., over-repetitive responding), and removed from analysis should these patterns be present. Additionally, of participants who completed all survey/task items in full, trial responses were checked for unusually short reaction times. Trials with reaction times below 200ms were excluded from data analysis as previous research indicates it takes 200 ms to respond to visual stimuli [35]. After excluding these trials, if participants had less than a 95% completion rate, they were excluded from data analysis.

### Statistical analysis

All data cleaning and statistical analyses were conducted in R [v4.2.2.; 36]. Pearson product moment correlations were performed on all continuous outcomes for the total sample and by biological sex to detect potential multicollinearity (not present for any predictor variable). Using scatterplots, we visually confirmed linear relationships between all continuous variables. For brevity, all correlation matrices are included as supplementary material. Correlations were performed using the HMisc (5.1-0) [37] and apaTables (2.0.8) [38] packages.

Multiple linear regression models (ordinary least squares) were conducted to examine whether handedness, age, biological sex, and the five AQ subscales (communication, attention switching, attention to detail, imagination) and their interaction with biological sex, predicted hemispheric bias. These were implemented as they allow us to estimate the relative and unique contribution of each predictor, as well as the flexibility to examine main and interaction effects. In model 1, we entered age, biological sex (reference = female, comparison = male), handedness, and all AQ subscale scores (social skills, communication, attention switching, attention to detail, and imagination), and their interaction with biological sex, as predictor variables with LQ1 (hemispheric bias based on choice) as the outcome variable. For model 2, we entered the same predictor variables as model 1 but with LQ2 (hemispheric bias based on reaction time) as the outcome variable. For model 1 and model 2, the assumptions of linearity (visual inspection of residual vs. fitted plots), non-autocorrelation (Durbin-Watson tests, all p’s > 0.05), and homoscedasticity (Breusch–Pagan, all p’s > 0.05) were met. Shapiro-Wilks tests were significant for model 1 and model 2 (both p’s < 0.05), however, visual inspection revealed the residual histogram plots were normally distributed [normality significance tests are overly sensitive with large samples; 39].

As the overall frequentist estimation of regression relationships (i.e., model 1 and model 2) were not statistically significant, we performed Bayes model averaging using the Bayesfactor package [0.9.12–4.5; 40] implemented in R [36] to calculate inclusion Bayes Factors (BF_Inclusion_). BF_Inclusion_ evaluates if the observed data are more probable in models with a specific predicator compared to models without that same predictor [41]. First, we built 10 linear models (ordinary least squares) including an intercepts-only model (*n* = 1), models with each individual predictor (*n* = 8), and a final model (*n* = 1) containing all individual predictors and interactions in model 1 and model 2. The bayes_factor_model function was used to calculate and compare Bayes factors (BF) for each model to the intercepts only model using BIC approximations [42, 43]. Finally, we used the bayesfactor_inclusion function (default settings; prior odds = uniform-equal; comparison = all models) to calculate BF_Inclusion_ which is the change from prior to posterior inclusion probabilities for each predictor and interaction effect. Kass and Raftery [44] empirically derived guidelines were used to interpret BF_Inclusion_ values (weak = 1–3; 3–20 = moderate; 20–150 = strong; > 150 = very strong).

## Results

### Descriptive statistics

Descriptive statistics for all continuous variables as a function of total sample and biological sex are presented in Table 2.

**Table 2:** Descriptive statistics for all continuous variables by total sample and biological sex

		Total					Male				Female		
Continuous variable	M	SD	Min	Max		M	SD	Min	Max	M	SD	Min	Max
Age	27.26	8.45	18.00	67.00		26.43	6.86	18.00	58.00	28.07	9.70	18.00	67.00
EHI	77.82	55.31	-100.00	100.00		75.15	57.73	-100.00	100.00	80.44	52.84	-100.00	100.00
AQ total	114.29	14.68	70.00	159.00		114.80	13.58	75.00	159.00	113.78	15.70	70.00	153.00
Social skills	22.84	5.07	10.00	37.00		22.39	4.93	10.00	37.00	23.29	5.17	12.00	36.00
Communication	20.88	4.90	10.00	38.00		21.26	4.59	10.00	34.00	20.51	5.16	10.00	38.00
Attention switching	25.20	4.26	13.00	37.00		25.22	3.77	14.00	36.00	25.18	4.70	13.00	37.00
Attention to detail	25.24	5.05	13.00	38.00		25.34	4.64	13.00	37.00	25.15	5.43	14.00	38.00
Imagination	20.12	3.89	10.00	31.00		20.59	3.57	11.00	31.00	19.65	4.13	10.00	30.00
LQ1 (choice bias)	0.17	0.32	-0.79	0.95		0.15	0.33	-0.79	0.86	0.19	0.31	-0.78	0.95
LQ2 (reaction time bias)	0.02	0.09	-0.39	0.34		0.01	0.09	-0.39	0.34	0.02	0.09	-0.32	0.30

Notes. N females = 214; N males = 210; N total = 424. AQ = autism spectrum quotient; EHI = Edinburgh Handedness Inventory; LQ1 = laterality quotient 1; LQ2 = laterality quotient 2

### Hemispheric selection bias (laterality quotient 1)

**Fig. 3:**
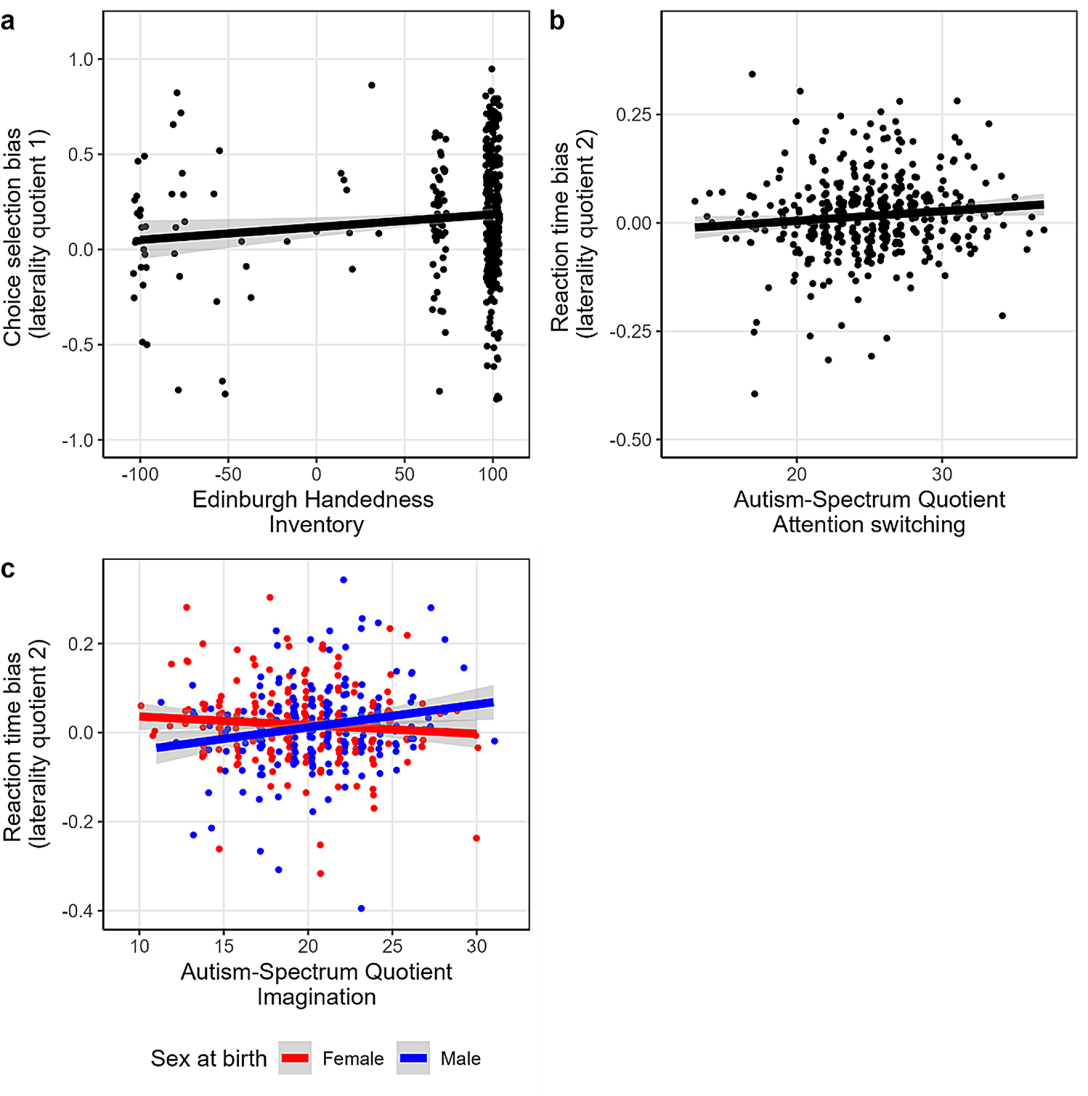
Scatterplots of significant regression findings. Note. Bivariate scatterplots, regression lines, and standard errors for (**a**) Edinburgh Handedness Inventory (EHI) and choice selection bias (laterality quotient 1); (**b**) Autism-Spectrum quotient – attention switching and reaction time bias (laterality quotient 2); and (**c**) Autism-Spectrum Quotient imagination*sex at birth interaction for reaction time bias (laterality quotient 2). All data points visually delineated by adding minor error/jitter

The first primary aim was tested in model 1, specifically, if handedness, age, biological sex, the five AQ subscales (social skills, communication, attention switching, attention to detail, and imagination) and their interactions with biological sex, significantly predicted LQ1 scores. Overall, model 1 was not statistically significant accounting for 4.50% of variance in LQ1 scores, *F*(13, 410) = 1.49, *p* = .119, adjusted R^2^ = 1.50%. Table 3 summarises the results. Handedness was the only significant positive predictor of LQ1 scores, *t*(410) = 2.70, *p* = .007, uniquely accounting for 1.69% of variance in LQ1 scores. More positive handedness scores (i.e., stronger right-handedness) were associated with stronger RHB based on choice. More negative handedness scores (i.e., stronger left-handedness) were associated with less choice bias. See Fig. 3a for scatterplot of handedness and laterality choice bias. No other predictors were statistically significant. BF_Inclusion_ analysis revealed weak support in favour of including handedness (BF_Inclusion_ = 2.331) with models containing handedness having an overall posterior inclusion probability of 36.8%. For all other predictors, BF_Inclusion_ values provided moderate (biological sex: BF_Inclusion_ = 1/6.00; posterior inclusion probability = 3.20%) to strong evidence (e.g., imagination*sex [male] interaction: BF_Inclusion_ = 1/1.84E + 12; posterior inclusion probability < 0.001%) against their inclusion. See Table 3 for BF_inclusion_ values.

**Table 3:** Summary of linear regression and bayesian estimates for LQ1 (hemispheric choice bias)

	Frequentist Estimates					Bayesian Estimates		
	Est.	SE	95% CI		*p*	*P*(incl.)	*P*(incl.|D)	BF_Inclusion_
Predictor			LL	UL				
(Intercept)	-0.017	-0.091	-0.391	0.356	0.928			
Handedness	0.001	2.698	0.000	0.001	**0.007**	0.20	0.368	2.331
Age	0.002	0.802	-0.002	0.005	0.423	0.20	0.032	0.131
Sex (male)^+^	0.034	0.121	-0.522	0.591	0.904	0.20	0.040	0.167
Social skills	0.001	0.240	-0.010	0.012	0.811	0.20	0.033	0.136
Communication	-0.005	-0.907	-0.017	0.006	0.365	0.20	0.026	0.106
Attention switching	0.011	1.921	0.000	0.022	0.055	0.20	0.035	0.146
Attention to detail	0.001	0.264	-0.007	0.009	0.792	0.20	0.024	0.098
Imagination	-0.006	-1.083	-0.017	0.005	0.279	0.20	0.022	0.089
Social Skills*Sex (male)	0.010	1.193	-0.007	0.027	0.233	0.10	< 0.001	< 0.001
Communication*Sex (male)	-0.009	-0.976	-0.027	0.009	0.330	0.10	< 0.001	< 0.001
Attention switching*Sex (male)	-0.011	-1.252	-0.029	0.006	0.211	0.10	< 0.001	< 0.001
Attention to detail*Sex (male)	-0.003	-0.434	-0.015	0.010	0.664	0.10	< 0.001	< 0.001
Imagination*Sex (male)	0.013	1.456	-0.004	0.029	0.146	0.10	< 0.001	< 0.001

Notes. ^+^Sex (reference group = female). Significant findings (*p* < .05) bolded. 95% CI = 95% confidence interval; est. = unstandardised regression coefficient; LL = lower limit; P(incl.) = prior inclusion probability; P(incl.|D) = posterior inclusion probability; SE = standard error; UL = upper limit

### Hemispheric reaction time bias (laterality quotient 2)

The second primary aim was evaluated in model 2, specifically, if handedness, age, biological sex, the five AQ subscales (social skills, communication, attention switching, attention to detail, and imagination) and their interaction with biological sex, significantly predicted LQ2 scores (hemispheric bias based on reaction time). Overall, model 2 was not statistically significant accounting for 4.81% of variance in LQ2 scores, *F*(13, 410) = 1.595, *p* = .083, adjusted R^2^ = 1.79%. Please refer to Table 4 for a summary. The attention switching subscale was a significant positive predictor of LQ2 scores (t[410] = 2.54, *p* = .012, sr^2^ = 1.5%). That is, individuals with higher AQ attention switching scores had stronger RHB (reaction time) than those with lower AQ attention switching scores. See Fig. 3b for attention switching and reaction time bias scatterplot. Imagination*biological sex (male) interaction (t[410] = 3.03, *p* = .003, sr^2^ = 2.1%) was a significant positive predictors of LQ2 scores, whereby males with higher AQ imagination scores exhibited a stronger RHB (reaction time) compared to males with lower AQ imagination scores. In contrast, females with higher AQ imagination scores exhibited a weaker RHB (reaction time) than females with lower AQ imagination scores. Refer to Fig. 3c for biological sex*imagination interaction scatterplot. No other predictors were statistically significant. BF_Inclusion_ analysis revealed weak support in favour of including the attention switching subscale (BF_Inclusion_ = 1.398) with models containing attention switching having an overall posterior inclusion probability of 25.9%. In contrast, there was strong evidence in favour of not including the imagination*biological sex (male) interaction (BF_Inclusion_ = 1/7.22E + 11; posterior inclusion probability < 0.001). For all other predictors, BF_Inclusion_ values provided moderate (e.g., imagination: BF_Inclusion_ = 1/6.35; posterior inclusion probability = 3.8%) to strong evidence (e.g., social skills*biological sex [male] interaction: BF_Inclusion_ = 1/7.22E + 11; posterior inclusion probability < 0.001%) against their inclusion. Please refer to Table 4 for all BF_inclusion_ values.

**Table 4:** Summary of linear regression and bayesian estimates for LQ2 (hemispheric reaction time bias)

	Frequentist Estimates					Bayesian Estimates		
	Est.	SE	95% CI		*p*	*P*(incl.)	*P*(incl.|D)	BF_Inclusion_
Predictor			LL	UL				
(Intercept)	-0.002	0.054	-0.108	0.104	0.972			
Handedness	< 0.001	< 0.001	< 0.001	< 0.001	0.491	0.20	0.028	0.115
Age	< 0.001	0.001	-0.001	0.001	0.787	0.20	0.026	0.106
Sex (male)^+^	-0.115	0.081	-0.274	0.043	0.152	0.20	0.027	0.110
Social skills	0.001	0.002	-0.003	0.004	0.708	0.20	0.029	0.119
Communication	-0.003	0.002	-0.006	0.001	0.126	0.20	0.031	0.129
Attention switching	0.004	0.002	0.001	0.007	**0.012**	0.20	0.259	1.398
Attention to detail	-0.001	0.001	-0.003	0.002	0.560	0.20	0.035	0.144
Imagination	-0.002	0.002	-0.005	0.001	0.258	0.20	0.038	0.158
Social skills*sex (male)	< 0.001	0.002	-0.005	0.005	0.982	0.10	< 0.001	< 0.001
Communication*sex (male)	< 0.001	0.003	-0.005	0.005	0.985	0.10	< 0.001	< 0.001
Attention switching*sex (male)	-0.002	0.003	-0.007	0.003	0.356	0.10	< 0.001	< 0.001
Attention to detail*sex (male)	0.001	0.002	-0.003	0.004	0.606	0.10	< 0.001	< 0.001
Imagination*sex (male)	0.007	0.002	0.003	0.012	**0.003**	0.10	< 0.001	< 0.001

Notes. ^+^Sex (reference group = female). Significant findings (*p* < .05) bolded. 95% CI = 95% confidence interval; est. = unstandardised regression coefficient; LL = lower limit; P(incl.) = prior inclusion probability; P(incl.|D) = posterior inclusion probability; SE = standard error; UL = upper limit

## Discussion

This study aimed to investigate the relationship between FEP hemispheric bias and relevant demographic and individual variables, including age, sex, handedness, and levels of autistic traits. To our knowledge, this study was the first to investigate these relationships in a sample this large and diverse from the general population, and to apply robust Bayesian modelling. The hypothesis that older people and females would demonstrate a reduced RHB for FEP was not supported. The hypothesis that left-handed people would demonstrate a reduced RHB for FEP was supported. Lastly, the hypotheses that individuals exhibiting higher levels of social and communication autistic traits would demonstrate a reduced RHB for FEP was not supported. Instead, frequentist statistical analyses suggested the AQ subscale score attention switching, and for males, the AQ subscale score imagination significantly predicted FEP hemispheric bias. However, it is important to note that Bayesian modelling did not support the inclusion of the imagination AQ subscale*biological sex interaction, and only provided weak support for the inclusion of the attention switching AQ subscale scores.

Similar to previous research conducted by Hellige et al. [15] and David [13], right-handed people tended to exhibit a greater RHB for FEP than left-handed people, as measured by Laterality Quotient 1 (based on the number of trials in which the participant chose the face that had the emotion presented in the left visual field, vs. the right visual field; LQ1). The present study extends on previous findings by investigating handedness continuously. These findings provide evidence for the involvement of handedness for hemispheric specialisation of cognitive processes, and specifically for FEP. Previous research conducted by Bourne [17] and Vladeanu et al. [10] both also examined handedness continuously, however found no effect of handedness on FEP hemispheric bias. Notably, these studies employed a much smaller sample than the present research, and it is possible that the small effect of handedness on FEP hemispheric bias was not captured in these studies. Evidence suggests that left-handed people may exhibit distinct hemispheric specialisation patters when compared to right-handed people [45, 46]. This finding extends to FEP, as evidenced by the present study, and motor and language processes [46–48]. While the cause of this association remains unclear, this suggests handedness provides insight regarding individual hemispheric specialisation patterns for various cognitive processes. It is possible that handedness reflects aspects of individual cytoarchitecture, and thus hemispheric specialisation.

Age and biological sex were not predictive of hemispheric bias in the present study. This conflicts with previous research conducted by Failla et al. [12], who reported that older people are more likely to have reduced RHB for FEP. Importantly, Failla et al. [12] examined this relationship categorically, and included those aged as young as 5–7 years, and as old as 60–70 years. It is possible that the age range where this relationship is evident was not captured in the present study. It is also possible that the distribution of ages in the present sample is not suitable for examining the relationship between age and FEP hemispheric bias. Our sample skews young (M = 27.23, 18–67), thus results are restricted primarily to young adults. The present findings also conflict with previous research conducted by Bourne [11], who found that females demonstrated a reduced RHB during FEP, and David [13] who found females had an increased RHB during FEP. This relationship was not evident in the present research. Given the interaction effect observed between sex and autistic traits in previous research conducted by Vladeanu et al. [10], it is possible that other cognitive and individual factors influence the relationship between sex and FEP hemispheric bias, explaining the heterogeneity of previous research. Indeed, we found an interaction between biological sex and the AQ subscale imagination, whereby males with higher AQ subscale imagination scores had higher Laterality Quotient 2 scores (i.e., the reaction time bias when selecting the more emotive face in the left or right visual fields; LQ2). This relationship was not present for females. However, frequentist modelling revealed that the sex (male)*AQ imagination interaction effect only accounted for 2.1% of variance in hemispheric reaction time bias. Moreover, complementary Bayesian modelling provided strong evidence against inclusion of this interaction term. The present study included a relatively large (*N* = 427) and diverse sample. Additionally, Bayesian modelling was used to evaluate the likelihood of the observed data in models with or without a given predictor. With these factors in mind, our findings do not provide evidence for the role of sex or age in hemispheric selection or reaction time bias. Large replication studies will help confirm or disconfirm these findings.

It was expected that the communication and social skill AQ subscales would be related to FEP hemispheric bias, however this was not evidenced by the present study. These findings do not align with that of previous research conducted by Brindley and Schmidt [8] and Taylor et al. [9], who suggested that autistic people are less right lateralised for FEP than neurotypical people. It is possible that the relationship between social and communication related autistic traits and FEP hemispheric bias is specifically related to a diagnosis of autism, and/or higher neurodivergence of autistics traits that were not captured in a sample of the general population. Evidence suggests that autistic individuals demonstrate distinct neural activation patterns that differ to that of neurotypical individuals [49, 50]. Specifically, during FEP, autistic individuals exhibit fusiform gyrus, amygdala, and superior temporal sulcus hypoactivation [49]. Along with this, electrophysiological evidence suggests autistic people exhibit atypical neural activation in response to facial stimuli [50]. This cortical activation variability may not be captured when examining autistic traits in the general population. Additionally, autistic individuals exhibit different gaze fixations during FEP than neurotypical individuals [8, 51–53]. Specifically, autistic individuals exhibit a significant right visual field bias during FEP, whereas neurotypical individuals exhibit a left visual field bias [8]. Autistic individuals are also less likely to fixate on the eyes when viewing emotional faces than neurotypical individuals [51, 53]. These gaze differences have previously been associated with cortical activation variability [51, 54, 55], and may not be captured when investigating the general population. These factors could explain the null findings regarding the relationship between FEP hemispheric bias and social and communication related autistic traits in the present research. Contrary to our hypothesis, autistic traits related to attention switching were related to FEP hemispheric reaction time bias (LQ2) such that higher levels of autistic traits were associated with a greater RHB, and lower levels of autistic traits related to attention switching were related to a greater LHB. That is, people with greater attention switching difficulties responded quicker when selecting the face with the emotion in the left visual field (and thus being processed by the right hemisphere first) than when selecting the face with the emotion presented in the right visual field (and thus being processed by the left hemisphere first). It is possible that FEP is more efficient when relevant information is processed in the right hemisphere for these individuals (as we anticipate for all typically developing individuals) and this is more prevalent/exacerbated by an inability to switch attention between visual fields.

Vladeanu et al. [10] previously reported that, for males, higher levels of autistic traits associated with social interest was related to a *stronger* RHB for FEP, and for females higher levels of autistic traits on this subscale was related to *reduced* RHB for FEP. In contrast, the present study found no main nor interaction effects of sex and levels of social/communication-related autistic traits on FEP hemispheric bias. One possible explanation for the conflicting findings between the present study and that of Vladeanu et al. [10] is the difference in measurement of autistic traits. While the present study used the AQ, Vladeanu et al. [10] used the broad autism phenotype questionnaire (BAPQ). The AQ subscales used here are specifically related to social and communication skills, whereas the aloof subscale in the BAPQ measures social interest and enjoyment [56]. This is notable as these subscales assess different aspects of social interactions. It is possible that the relationship between social/communication skills and FEP hemispheric bias, and social interest and enjoyment and FEP hemispheric bias may be different. Further, there is some evidence to suggest that the sex differences in FEP hemispheric bias are driven by hormonal (and specifically testosterone) differences [57]. Compared to the present study Vladeanu et al. [10] employed a smaller, and less diverse sample, reducing the generalisability of their findings.

Previous research suggests that hemispheric bias for FEP may be dependent on the emotion being observed [3]. There are several theories proposing different relationships between observed emotion and FEP hemispheric bias which may explain the lack of associations observed in the present study. The present research provides foundational evidence for the role of various individual factors in FEP hemispheric bias, and future research should aim to extend on this by examining the role of emotion.

Limiting the findings of the present study was the use of online testing. As a result, we were restricted to offsite data collection. Our measurement of FEP hemispheric bias using a Chimeric Faces Task, while used previously and validated elsewhere [7, 17, 32, 33] is considered a proxy for measurement of underlying cortical activation. Future research would benefit from directly measuring hemispheric bias by way of neuroimaging techniques (e.g., functional magnetic resonance imaging). Further, there are several other techniques that should be utilised in future research that could not be used here due to the limitations of online research. This includes eye tracking, which could be used to verify participant fixations and hemi-spatial bias [51, 52, 58], and other neuroimaging and electrophysiological (e.g., electroencephalography) techniques to characterise the neural substrates of hemispheric facial processing bias [49, 54, 58]. Finally, the present study is subject to other limitations associated with online research. There were likely to be variations in the testing environment between participants which is uncontrollable, and there is potential for data contamination due to inattentive responding which likely increases noise. While relevant steps were taken to identify and exclude such responses, these potential confounds are inherent limitations in online research.

With consideration of previous research, the results from the present study provide evidence that the relationship between autistic traits and FEP hemispheric bias is nuanced and should be investigated further in future research using both clinical and non-clinical samples. Additionally, handedness and autistic traits related to attention were significant predictors of FEP hemispheric bias, alongside weak support for autistic traits related to imagination predicting FEP hemispheric biases in males, but not females. Future research ought to consider the effects of observed emotion on hemispheric bias of FEP and would benefit from more direct measures of FEP hemispheric bias.

## Electronic supplementary material

Below is the link to the electronic supplementary material.

Supplementary Material 1

## Data Availability

Data available from corresponding author on reasonable request.

## Abbreviations

AQ: Autism-spectrum Quotient
BAPQ: The broad autism phenotype questionnaire
BF: Bayes factor
BF_inclusion_: Inclusion bayes factor
EHI: Edinburgh Handedness Inventory
FEP: Facial emotion processing
LHB: Left-hemispheric bias
LQ: Laterality quotient
RADIATE face stimulus set: The racially diverse affective expression face stimulus set
RHB: Right-hemispheric bias
