# Supplementary material for: Facial emotion processing hemispheric bias is weakly associated with handedness, autistic traits and biological sex, but not age
Source: BMC Psychol. 2025 Mar 18;13:271. doi: 10.1186/s40359-024-02218-2 (PMC11921578; doi:10.1186/s40359-024-02218-2)
Supplement: Supplementary file 1 — Supplementary Material 1 [file 40359_2024_2218_MOESM1_ESM.docx]

**Supplementary Materials**

**Supplementary Table 1**

For the total sample, Autism-Spectrum quotient (AQ) total scores had moderate to strong significant correlations with social skills, attention switching, attention to detail, communication, and imagination. Age had weak negative significant correlations with AQ total scores, attention switching, attention to detail, and communication. Most AQ subscales had weak to moderate significant positive correlations with the other AQ subscales. Laterality quotient 1 (LQ1; hemispheric choice bias) had a weak positive significant correlation with the Edinburgh Handedness Inventory (EHI) and a strong significant positive correlation with the laterality quotient 2 (LQ2; reaction time bias). Please refer to Supplementary Table 1 for all correlations amongst continuous variables.

**Supplementary Table 1**

*Pearson product moment correlations for all continuous variables for the total sample*

| Variable | 1 | 2 | 3 | 4 | 5 | 6 | 7 | 8 | 9 | 10 |
| --- | --- | --- | --- | --- | --- | --- | --- | --- | --- | --- |
| 1. Age | - |  |  |  |  |  |  |  |  |  |
|  |  |  |  |  |  |  |  |  |  |  |
| 2. AQ total score | **-.13**** | - |  |  |  |  |  |  |  |  |
|  | [-.22, -.03] |  |  |  |  |  |  |  |  |  |
| 3. Social skills | 0.02 | **.77**** | - |  |  |  |  |  |  |  |
|  | [-.08, .11] | [.72, .80] |  |  |  |  |  |  |  |  |
| 4. Attention | **-.18**** | **.68**** | **.48**** | - |  |  |  |  |  |  |
| switching | [-.27, -.09] | [.62, .73] | [.40, .55] |  |  |  |  |  |  |  |
| 5. Attention to detail | -0.06 | **.37**** | -0.02 | 0.01 | - |  |  |  |  |  |
|  | [-.15, .04] | [.29, .45] | [-.12, .07] | [-.08, .11] |  |  |  |  |  |  |
| 6. Communication | **-.16**** | **.82**** | **.65**** | **.52**** | 0.09 | - |  |  |  |  |
|  | [-.25, -.07] | [.78, .85] | [.59, .70] | [.44, .58] | [-.01, .18] |  |  |  |  |  |
| 7. Imagination | -0.03 | **.52**** | **.28**** | **.17**** | 0.01 | **.31**** | - |  |  |  |
|  | [-.13, .06] | [.44, .58] | [.19, .36] | [.07, .26] | [-.09, .11] | [.22, .40] |  |  |  |  |
| 8. EHI | -0.04 | -0.06 | -0.08 | -0.05 | -0.04 | -0.04 | 0.02 | - |  |  |
|  | [-.14, .05] | [-.16, .03] | [-.17, .02] | [-.14, .05] | [-.14, .05] | [-.13, .06] | [-.08, .11] |  |  |  |
| 9. LQ1 | 0.05 | 0 | 0.03 | 0.05 | -0.03 | -0.05 | -0.02 | **.12*** | - |  |
| (choice bias) | [-.05, .14] | [-.10, .09] | [-.06, .13] | [-.04, .15] | [-.12, .07] | [-.14, .05] | [-.11, .08] | [.02, .21] |  |  |
| 10. LQ2 | 0.01 | 0.03 | 0.03 | **.10*** | -0.04 | -0.02 | 0.04 | 0.02 | **.58**** | - |
| (reaction time bias) | [-.09, .10] | [-.06, .13] | [-.07, .13] | [.01, .20] | [-.13, .06] | [-.12, .07] | [-.05, .14] | [-.07, .12] | [.51, .64] |  |

*Notes.* N total = 424. Significant results bolded (*p* < .05). 95% confidence intervals contained in square brackets. AQ = autism spectrum quotient; EHI = Edinburgh Handedness Inventory; LQ1 = laterality quotient 1; LQ2 = laterality quotient 2.

**Supplementary Table 2**

For males, AQ total scores had moderate to strong significant correlations with social skills, attention switching, attention to detail, communication and imagination. Most AQ subscales showed moderate to strong positive correlations with the other AQ subscales. Age had weak negative significant correlations with AQ total scores, attention switching, communication, and the EHI. LQ1 (hemispheric choice bias) had a weak positive significant correlation with EHI and a strong positive significant correlation with LQ2 (reaction time bias). Please refer to Supplementary Table 2 for all correlations amongst continuous variables for males (lower diagonal).

For females, AQ total scores had moderate to strong significant correlations with social skills, attention switching, attention to detail, communication, and imagination. Most AQ subscales had moderate to strong positive significant correlations with the other AQ subscales. Age had a weak negative significant correlation with attention switching. LQ2 (reaction time bias) had a weak positive significant correlation with attention switching and a strong positive significant correlation with LQ1 (choice bias). Please refer to Supplementary Table 2 for all correlations amongst continuous variables for females (upper diagonal).

**Supplementary Table 2**

Pearson product moment correlations for all continuous variables as a function of biological sex: males (lower diagonal), females (upper diagonal)

| Variable | 1 | 2 | 3 | 4 | 5 | 6 | 7 | 8 | 9 | 10 |
| --- | --- | --- | --- | --- | --- | --- | --- | --- | --- | --- |
| 1. Age | - | -0.10 | 0.02 | **-.18**** | -0.05 | -0.12 | -0.01 | 0.02 | 0.02 | 0.05 |
|  |  | [-.23, .03] | [-.11, .16] | [-.30, -.04] | [-.18, .09] | [-.25, .02] | [-.14, .12] | [-.11, .16] | [-.12, .15] | [-.08, .19] |
| 2. AQ total score | **-.16*** | - | **.76**** | **.67**** | **.44**** | **.81**** | **.50**** | -0.01 | 0.02 | -0.01 |
|  | [-.29, -.03] |  | [.70, .81] | [.59, .74] | [.32, .54] | [.75, .85] | [.40, .60] | [-.15, .12] | [-.12, .15] | [-.15, .12] |
| 3. Social skills | -0.02 | .79** | - | **.46**** | **0.04** | **.63**** | **.27**** | -0.02 | 0.02 | 0.01 |
|  | [-.15, .12] | [.74, .84] |  | [.34, .56] | [-.09, .17] | [.54, .70] | [.14, .39] | [-.15, .12] | [-.12, .15] | [-.12, .15] |
| 4. Attention | **-.19**** | **.69**** | **.52**** | - | 0.06 | **.52**** | 0.12 | -0.06 | 0.10 | **.14*** |
| switching | [-.32, -.05] | [.61, .75] | [.41, .61] |  | [-.08, .19] | [.41, .61] | [-.02, .25] | [-.19, .08] | [-.03, .23] | [.00, .27] |
| 5. Attention to detail | -0.07 | **.28**** | -0.09 | -0.05 | - | 0.12 | 0.08 | -0.01 | 0.01 | -0.05 |
|  | [-.20, .07] | [.15, .40] | [-.23, .04] | [-.18, .09] |  | [-.01, .25] | [-.06, .21] | [-.14, .13] | [-.13, .14] | [-.19, .08] |
| 6. Communication | **-.21**** | **.84**** | **.69**** | **.52**** | 0.04 | - | **.27**** | -0.03 | -0.02 | -0.05 |
|  | [-.34, -.08] | [.79, .87] | [.61, .76] | [.41, .61] | [-.10, .17] |  | [.14, .39] | [-.16, .11] | [-.15, .12] | [-.18, .09] |
| 7. Imagination | **-0.04** | **.54**** | **.32**** | **.25**** | -0.09 | **.35**** | - | 0.08 | -0.07 | -0.09 |
|  | [-.18, .09] | [.43, .63] | [.19, .44] | [.11, .37] | [-.22, .05] | [.22, .46] |  | [-.05, .21] | [-.20, .07] | [-.22, .04] |
| 8. EHI | **-.14*** | -0.12 | **-.15*** | -0.04 | -0.08 | -0.04 | -0.04 | - | 0.06 | -0.03 |
|  | [-.27, -.01] | [-.25, .02] | [-.28, -.01] | [-.17, .10] | [-.22, .05] | [-.17, .10] | [-.17, .10] |  | [-.08, .19] | [-.17, .10] |
| 9. LQ1 | 0.07 | -0.02 | 0.04 | -0.01 | -0.07 | -0.07 | 0.05 | **.17*** | - | **.54**** |
| (choice bias) | [-.06, .21] | [-.16, .11] | [-.10, .17] | [-.15, .13] | [-.20, .07] | [-.20, .07] | [-.08, .19] | [.03, .29] |  | [.43, .63] |
| 10. LQ2 | -0.06 | 0.08 | 0.04 | 0.07 | -0.02 | 0.00 | **.20**** | 0.06 | **.61**** | - |
| (reaction time bias) | [-.19, .08] | [-.06, .21] | [-.09, .18] | [-.07, .20] | [-.16, .11] | [-.13, .14] | [.06, .32] | [-.07, .20] | [.52, .69] |  |

Notes. N females = 214; N males = 210. Significant results bolded (*p* < .05). 95% confidence intervals contained in square brackets. AQ = autism spectrum quotient; EHI = Edinburgh Handedness Inventory; LQ1 = laterality quotient 1; LQ2 = laterality quotient 2.
